# N-Type Mg_3_Sb_2-*x*_Bi*_x_* Alloys as Promising Thermoelectric Materials

**DOI:** 10.34133/2020/1219461

**Published:** 2020-11-25

**Authors:** Hongjing Shang, Zhongxin Liang, Congcong Xu, Jun Mao, Hongwei Gu, Fazhu Ding, Zhifeng Ren

**Affiliations:** ^1^Department of Physics and Texas Center for Superconductivity at the University of Houston (TcSUH), University of Houston, Houston, TX 77204, USA; ^2^Key Laboratory of Applied Superconductivity and Institute of Electrical Engineering, Chinese Academy of Sciences, Beijing 100190, China; ^3^University of Chinese Academy of Sciences, Beijing 100049, China

## Abstract

N-type Mg_3_Sb_2-*x*_Bi*_x_* alloys have been extensively studied in recent years due to their significantly enhanced thermoelectric figure of merit (*zT*), thus promoting them as potential candidates for waste heat recovery and cooling applications. In this review, the effects resulting from alloying Mg_3_Bi_2_ with Mg_3_Sb_2_, including narrowed bandgap, decreased effective mass, and increased carrier mobility, are summarized. Subsequently, defect-controlled electrical properties in n-type Mg_3_Sb_2-*x*_Bi*_x_* are revealed. On one hand, manipulation of intrinsic and extrinsic defects can achieve optimal carrier concentration. On the other hand, Mg vacancies dominate carrier-scattering mechanisms (ionized impurity scattering and grain boundary scattering). Both aspects are discussed for Mg_3_Sb_2-*x*_Bi*_x_* thermoelectric materials. Finally, we review the present status of, and future outlook for, these materials in power generation and cooling applications.

## 1. Introduction

Thermoelectric technology, which can achieve reversible conversion between electricity and heat, holds great potential for alleviating the energy and environmental crises [1, 2]. However, large-scale commercialization of thermoelectric technology has yet to be implemented, mainly due to the low energy-conversion efficiency of existing thermoelectric materials. The thermoelectric energy-conversion efficiency is contingent on the materials' dimensionless figure of merit *zT* = *S*^2^*σT*/(*κ*_*e*_ + *κ*_l_), where *S* is the Seebeck coefficient, *σ* is the electrical conductivity, *T* is the absolute temperature, *κ*_*e*_ is electronic thermal conductivity, and *κ*_*l*_ is the lattice thermal conductivity [3–6].

Currently, advancements have been achieved in many kinds of thermoelectric materials, such as lead chalcogenides [7, 8], SnSe [9–11], and half-Heuslers [12, 13] at medium and high temperatures. However, progress on near-room-temperature materials has been sluggish. The Bi_2_Te_3_-based compounds, discovered in the 1950s, have remained the state-of-the-art thermoelectric materials at around room temperature for several decades [14, 15]. However, these materials are still not widely applied in viable thermoelectric applications due to the high cost of tellurium (Te) and some unresolved engineering issues (e.g., high contact resistance between the contact materials and the thermoelectric legs when nanostructured materials are considered for making the modules).

**Figure 1:**
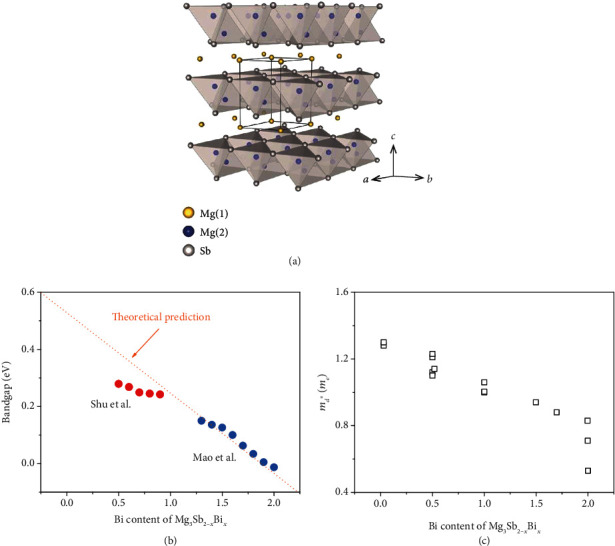
(a) Crystal structure of Mg_3_Sb_2_. Reproduced with permission from Ref. [17]. Copyright 2016 John Wiley and Sons. (b) Bandgap energy of Mg_3_Sb_2-*x*_Bi*_x_* as a function of composition [23, 32]. (c) Density of state effective mass (*m*_d_^∗^) for n-type Mg_3_Sb_2*-x*_Bi*_x_* as a function of composition [23, 28, 34, 35].

Recently, the n-type Mg_3_Sb_2-*x*_Bi*_x_* alloys have attracted significant attention because of their promising thermoelectric performance and good mechanical properties, the abundance and low cost of their constituent elements, etc. Mg_3_Sb_2_ has a CaAl_2_Si_2_-type crystal structure, which consists of an octahedrally coordinated cation Mg^2+^ layer and a tetrahedrally coordinated anion structure (Mg_2_Sb_2_)^2-^ that form a nearly isotropic three-dimensional (3D) chemical bonding network with an interlayer bond that is mostly ionic and partially covalent (Figure 1(a)) [16]. These crystallographic characteristics lead to decent electrical properties, intrinsically low lattice thermal conductivity, and good mechanical properties. Actually, Mg_3_Sb_2-*x*_Bi*_x_* alloys have long been regarded as persistent p-type semiconductors, and their n-type counterparts were considered to be impossible to synthesize, which should be attributed to the negatively charged Mg vacancies that pin the Fermi level around the valence band [17–19]. This was the case until n-type Mg_3_Sb_2-*x*_Bi*_x_* with high thermoelectric performance was reported by Tamaki et al. [17] through the addition of excess Mg and doping with Te, although Zhang et al. [20] soon after reported similar results with Te doping only. The extra Mg can effectively suppress the Mg vacancies, thus rendering n-type conduction in Mg_3_Sb_2-*x*_Bi*_x_* [17, 21, 22]. Since the discovery of n-type Mg_3_Sb_2-*x*_Bi*_x_*, notable advancements have been made, and its state-of-the-art average *zT* has been raised up to ~1.1 in the range of 300-500 K, comparable to that of the Bi_2_Te_3_-based materials [23–29].

This review focuses on these n-type Mg_3_Sb_2-*x*_Bi*_x_* alloys with promising thermoelectric performance. We first summarize the effects of alloying Mg_3_Sb_2_ with Mg_3_Bi_2_ on the band structure (e.g., bandgap, effective mass, and carrier mobility). The defect-controlled electronic transport in Mg_3_Sb_2-*x*_Bi*_x_* thermoelectric materials will then be discussed, including defect-chemistry-inspired dopant exploration and the defect-induced near-room-temperature shift in the carrier-scattering mechanism. Furthermore, promising applications in power generation and cooling are also discussed. The strategies mentioned here are believed to be equally applicable to many other thermoelectric materials. Some ideas for possible further improvement of thermoelectric performance in n-type Mg_3_Sb_2-*x*_Bi*_x_* materials are also presented.

## 2. Electronic Structure

Alloying of Mg_3_Sb_2_ with Mg_3_Bi_2_ has a significant impact on the thermoelectric transport properties and band structures of the alloys. Zhang et al. [30] calculated the band alignments of Mg_3_Sb_2-*x*_Bi*_x_* alloys and found that Mg_3_Bi_2_ alloying results in a moderate increase in the energy separation between the conduction band minima K and CB_1_, decreasing the contribution of the secondary band minimum K to the electrical transport. Since Mg_3_Bi_2_ is a semimetal [31] and Mg_3_Sb_2_ is a semiconductor, the bandgap of Mg_3_Sb_2-*x*_Bi*_x_* will be reduced with increasing Mg_3_Bi_2_ content (Figure 1(b)), leading to an enhanced bipolar contribution for the Bi-rich compositions [23, 32]. Thus, such compositions are not suitable for applications at higher temperatures. Considering the empirical trend of bandgap dependence on the application temperature range, the room temperature thermoelectric materials exhibit similar bandgaps, so the bandgap of Bi_2_Te_3-*x*_Se*_x_* provides a hint for choosing Mg_3_Sb_2-*x*_Bi*_x_* compositions with the proper Bi/Sb ratios [32].

In addition, the effective mass will be reduced with increasing Mg_3_Bi_2_ concentration [31]. Theoretically, with increasing Bi content in Mg_3_Sb_2-*x*_Bi*_x_*, the density of states effective mass (*m*_*d*_^∗^) is reduced from ~1.53 *m*_0_ (Mg_3_Sb_2_) to ~1.23 *m*_0_ (Mg_3_SbBi) to ~0.87 *m*_0_ (Mg_3_Bi_2_) based on the simulation from the BoltzTraP software package with spin orbit coupling (SOC) (300 K, carrier concentration: ~4 × 10^19^ cm^−3^), leading to a smaller Seebeck coefficient and higher carrier mobility [31]. Such a trend has been verified experimentally although the values seem to be lower than the theoretical calculation, as shown in Figure 1(c). It is clear that Bi alloying significantly reduces the density of states effective mass, indicating that it is an effective strategy to enhance the carrier mobility of Mg_3_Sb_2-*x*_Bi*_x_* alloys. Therefore, the alloying concentration of Mg_3_Bi_2_ is crucial for balancing the carrier mobility and the Seebeck coefficient, as well as the bipolar effect. Pan et al. [33] showed the band evolution from Mg_3_Bi_2_ to Mg_3_Sb_2_ through angle-resolved photoemission spectroscopy (ARPES) combined with density functional theory (DFT) calculations, which also indicated the effectiveness of adjusting the Bi/Sb ratio in improving thermoelectric performance.

## 3. Chemical Doping

Defect chemistry has been widely investigated in thermoelectric Zintl compounds in order to understand their intrinsic defects and to explore effective extrinsic dopants that can optimize their electronic transport properties [36–38]. In Mg_3_Sb_2-*x*_Bi*_x_* alloys, native Mg vacancies caused by the low defect formation energy and high vapor pressure of Mg result in p-type conduction and abnormal electronic transport behavior near room temperature. Recent studies have shown that adding excess Mg could suppress the formation of such vacancies, leading to a reduction in hole concentration and further resulting in n-type conduction behavior [22]. However, due to the intrinsic doping limit, the electron concentration achieved is only ~10^18^ cm^−3^, which is significantly lower than the optimal carrier concentration (~10^19^ cm^−3^) needed to maximize the *zT*. Thus, further optimization of the electron concentration *via* extrinsic doping at the Mg or Sb/Bi sites is especially necessary in this case.

**Figure 2:**
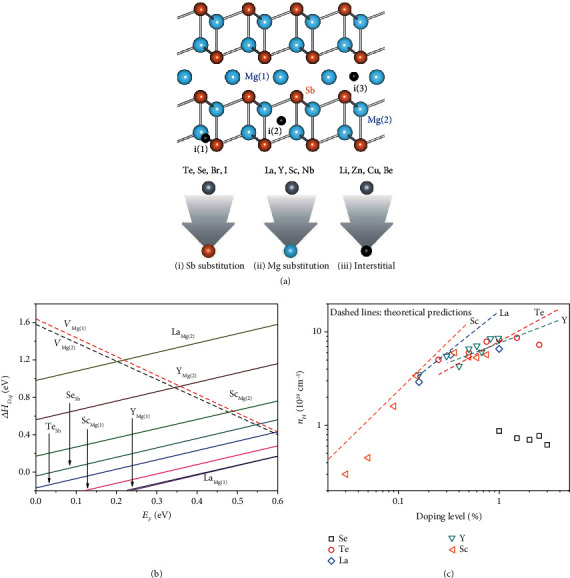
(a) Mg_3_Sb_2_ contains two unique Mg Wyckoff positions denoted by Mg(1) and Mg(2) and one unique Sb Wyckoff position. Reproduced with permission from Ref. [40]. Copyright 2018 Royal Society of Chemistry. (b) Defect formation energy (Δ*H*_*D*,*q*_) of various dopants as a function of the Fermi energy (*E*_*F*_) under the Mg-rich condition [39, 40]. (c) Doping efficiency of some dopants (Te, Se, La, Y, Sc) in Mg_3_Sb_2-*x*_Bi*_x_* at 300 K, with a comparison to ideal doping (dashed lines) assuming that each donor releases one electron [27, 34, 42, 45, 49].

Gorai et al. [39, 40] used first principle defect calculations to study n-type doping strategies for Mg_3_Sb_2-*x*_Bi*_x_* alloys, including (i) Sb substitution by mono- (Br, I) or divalent (Se, Te) anions, (ii) Mg substitution by trivalent or higher valence cations (La, Y, Sc, Nb), and (iii) insertion of cation interstitials (Li, Zn, Cu, Be), which are represented by black spheres and denoted by i(1), i(2), and i(3) in Figure 2(a). The chemical trends of various dopants have been revealed in terms of their solubility and maximum achievable electron concentration, and the discussion here mainly focuses on Sb and Mg substitution. For the Sb substitution strategy, the defect formation energy around the conductive band minimum in Te_Sb_ is lower than that in Se_Sb_ under the Mg-rich condition (Figure 2(b)), indicating that Te may have a higher doping limit and greater efficiency, both of which have been confirmed experimentally [20, 35, 41]. On the other hand, substitution by La, Y, and Sc at the cation site has been also explored. It has been found that the defect formation energy values of La_Mg(1)_, Y_Mg(1)_, and Sc_Mg(1)_ are each lower than that of Te_Sb_, indicating that Mg substitution is even more effective than Sb substitution by Se or Te. The predicted carrier concentration in (La, Y, Sc)-doped Mg_3_Sb_2_ could exceed ~10^20^ cm^−3^. The relationship between the dopant concentration and the measured electron concentration of Mg_3_Sb_2-*x*_Bi*_x_* for different dopants, i.e., La [42], Y [43], Sc [34], Se [35, 44], and Te [45], is illustrated in Figure 2(c). For each dopant, the carrier concentration gradually saturates at a given value with increasing doping level, which is slightly different from the theoretical predictions (dashed lines). This may be closely related to the limited solubility of dopants in Mg_3_Sb_2-*x*_Bi*_x_* alloys. Additionally, the optimized carrier concentration for power generation is in the range of ~3 − 5 × 10^19^ cm^−3^, and it is slightly lower for cooling, and such carrier concentrations can be achieved by doping with Te, Y, Sc, and La. Actually, most studies reported thus far have focused on how to improve the *zT* value, ignoring the structural origin: e.g., how the electronic and atomic structures of the alloys, including the chemical bonding and the chemical state, evolve after introducing the dopant; how the band structures vary due to doping; and whether a chemical reaction occurs at high temperature. Such lack of structural understanding limits further improvement in the thermoelectric performance of the Mg_3_Sb_2-*x*_Bi*_x_* alloys.

Additionally, it should be noted that dopants may affect the thermal stability of the n-type Mg_3_Sb_2-*x*_Bi*_x_* alloys, with studies suggesting that degradation in performance would occur with their long-term operation at high temperatures (≥673 K) and that cation-site doping (Y, La, Yb, etc.) *via* replacing excess Mg may improve their thermal stability and delay such decline in the thermoelectric properties [42, 46, 47]. This can be explained by the changing defect energetics and the fewer Mg deficiencies. Considering the differences in vapor pressure between Mg and Bi/Sb, the decreasing thermal stability has been attributed to the significant Mg loss (defects) at high temperature [48]. Cation-site doping can effectively eliminate Mg deficiencies and improve the thermal stability. On the other hand, by applying coating (such as boron nitride, etc.) on the surfaces of the Mg_3_Sb_2-*x*_Bi*_x_* alloys, their thermal stability can be also effectively improved since such coating prevents Mg loss. Thus, both cation-site doping and coating technology are beneficial for improving thermal stability and promoting practical applications, especially power generation at elevated temperatures.

## 4. Manipulating the Carrier-Scattering Mechanism

**Figure 3:**
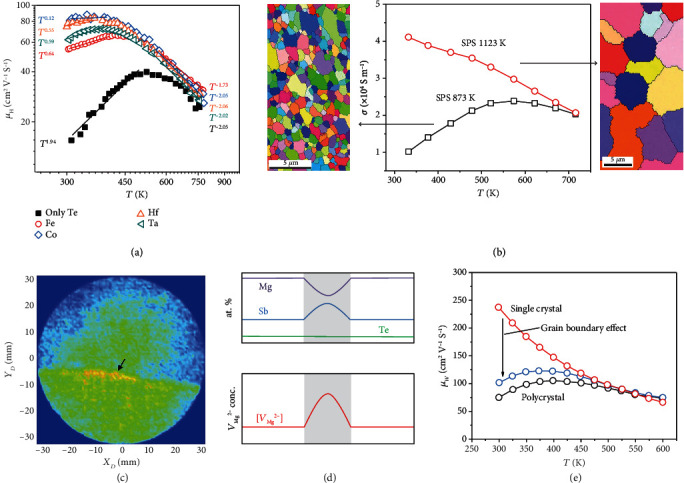
Electronic properties and structures of Mg_3_Sb_2-*x*_Bi*_x_*. (a) Temperature-dependent Hall mobility [25]. (b) Temperature-dependent electrical conductivity and electron backscatter diffraction (EBSD) crystal orientation maps. Reproduced with permission from Ref. [53]. Copyright 2018 American Institute of Physics. (c) Projected atomic density map from 3D APT measurement showing the planar Mg-deficiency defect (arrow); (d) such Mg deficiency in the grain boundary region (gray area, top panel) induces a higher Mg vacancy (*V*_Mg_^2-^) concentration (bottom panel). Reproduced with permission from Ref. [56]. Copyright 2019 John Wiley and Sons. (e) Temperature-dependent weighted mobility [57]. Reproduced with permission from Ref. 50. Copyright 2020 John Wiley and Sons.

In addition to tuning the carrier concentration, suppression of Mg vacancies in n-type Mg_3_Sb_2-*x*_Bi*_x_* could also be employed to manipulate the carrier-scattering mechanism, thereby enhancing carrier mobility and improving the *zT*, which is particularly significant near room temperature. By exploring the Hall carrier mobility (*μ_H_*) temperature (*T*) relation, ionized impurity scattering was found to dominate the electron transport around room temperature, resulting in low carrier mobility [45]. In order to reduce Mg vacancies and suppress ionized impurity scattering in Mg_3.2_Sb_1.5_Bi_0.49_Te_0.01_, Mao et al. [25] introduced transition-metal elements (Fe, Co, Hf, Ta) into the material matrix, eventually increasing the room-temperature carrier mobility from ~16 cm^2^ V^−1^ s^−2^ to ~81 cm^2^ V^−1^ s^−2^ (Figure 3(a)). Similarly, other transition-metal elements, such as Nb [24] and Mn [5, 32, 44], have also been shown to have a dominant effect in shifting the scattering mechanism from ionized impurity scattering to a mixture of ionized impurity scattering and acoustic phonon scattering around room temperature. Additionally, since defects are highly sensitive to preparation conditions, Mao et al. [50] reported that manipulating the hot-pressing temperature could also tune the carrier-scattering mechanism and thereby substantially enhance the carrier mobility of Mg_3.2_Sb_1.5_Bi_0.49_Te_0.01_.

On the other hand, grain boundary scattering has also attracted increasing attention as a carrier-scattering mechanism other than ionized impurity scattering because samples with large grain size have been shown to demonstrate higher carrier mobility, which is particularly noticeable around room temperature [51, 52]. The Mg_3.2_Sb_1.5_Bi_0.49_Te_0.01_ samples prepared at a higher sintering temperature show noticeably enlarged grain size as well as higher electrical conductivity (Figure 3(b)). For example, the room-temperature electrical conductivity is ~4 × 10^4^ S m^−1^ for the sample with an average grain size of ~7.8 *μ*m, and it is ~1 × 10^4^ S m^−1^ for the sample with an average grain size of ~1.0 *μ*m [53]. Similarly, the grain size of Mg_3_Sb_2-*x*_Bi*_x_* alloys was increased by annealing [54] or hot deforming [27, 34, 55], and improvement in mobility was also observed. It should be noted that the defects would be also reduced, in addition to the increasing grain size, by increasing the sintering temperature or by annealing. Thus, in these cases, the ionized impurity scattering was also reduced, eventually leading to the increased electrical conductivity. Kuo et al. explored the defect compositions near the grain boundary of Mg_3.05_Sb_1.99_Te_0.01_ (nominal composition) using 3D atom-probe tomography (APT) (Figure 3(c)), from which the planar defect is clearly noticeable (as marked by the arrow), and it is a maximum 5 at. % Mg deficiency [56]. As discussed above, a Mg deficiency could easily induce a high Mg vacancy (*V*_Mg_^2-^) concentration in the vicinity of the boundary and result in the depletion of free n-type carriers since *V*_Mg_^2-^ serves as an effective electron-killing defect (Figure 3(d)). Single-crystal n-type Mg_3_Sb_2_ was thus grown and used to investigate the underlying charge-scattering mechanism [33, 57, 58]. As indicated in Figure 3(e), acoustic phonon scattering dominates the charge transport in the single-crystal sample that lacks grain boundary electrical resistance, resulting in the sample's significantly increased weighted mobility near room temperature. This may support the proposition that grain boundary scattering dominates the carrier transport of n-type Mg_3_Sb_2-*x*_Bi*_x_* alloys in the near-room-temperature range but does not exclude the ionized impurity scattering existing in the samples that do have lots of defects. Actually, in comparison to polycrystal Mg_3_Sb_2-*x*_Bi*_x_*, not only grain boundaries but also defects are reduced in the single-crystal sample. Thus, additional details are needed to clarify the carrier-scattering mechanism, which is also crucial for further improving the thermoelectric performance of n-type Mg_3_Sb_2-*x*_Bi*_x_*.

## 5. Power Generation and Cooling Applications

Mg_3_Sb_2-*x*_Bi*_x_* alloys have shown promise for applications in power generation and cooling due to their high performance. Generally, the Sb-rich compositions (Mg_3_Sb_2_-based alloys) are promising for power generation at medium temperature although they may lack good stability due to Mg loss at high temperature (≥673 K). For example, Zhu et al. [59] reported that the conversion efficiency of Mg_3.1_Co_0.1_Sb_1.5_ Bi_0.49_Te_0.01_ could be up to ~10.6% at a temperature difference of 400 K in the range from 300 K to 700 K, suggesting good potential for midtemperature heat conversion.

The Bi-rich compositions (Mg_3_Bi_2_-based materials), on the other hand, show more potential for cooling applications. In this case, concerns regarding thermal stability can be ignored due to the low temperature range. Mao et al. [23] reported that optimized Mg_3.2_Sb_0.5_Bi_1.498_Te_0.02_ exhibits a room temperature *zT* of more than 0.7 and that the unicouple of Mg_3.2_Sb_0.5_Bi_1.498_Te_0.02_ and Bi_0.5_Sb_1.5_Te_3_ exhibits a large temperature difference of ~91 K at the hot-side temperature of 350 K, comparable to that of commercial coolers based on the Bi_2_Te_3_ alloys. Imasato et al. [26] also fabricated n-type Mg_3_Sb_0.6_Bi_1.4_ with a *zT* of 1.0-1.2 at 400-500 K, which surpasses that of the n-type Bi_2_Te_3_. Furthermore, Mg_3_Sb_2-*x*_Bi*_x_* alloys are inexpensive compared to Bi_2_Te_3_-based materials because they minimize the need for expensive elemental Te, largely reducing the material cost. In addition, unlike the nanostructured n-type Bi_2_Te_3_-based materials that suffer from high contact resistance between the thermoelectric legs and the electrodes, such contact resistance can be greatly reduced for Mg_3_Sb_2-*x*_Bi*_x_* by forming a sandwiched structure of Fe/Mg_3_Sb_2-*x*_Bi*_x_*/Fe. All of these examples show the great potential that the Mg_3_Sb_2-*x*_Bi*_x_* alloys have for becoming good candidates to replace the traditional Bi_2_Te_3_, promoting their application in thermoelectric technology. In particular, the high cooling performance of Mg_3_Bi_2_-based alloys inspires researchers to explore these semimetals as potential thermoelectric materials for cooling.

## 6. Conclusions

In summary, strategies like alloying, as well as defect-controlled carrier-concentration optimization and manipulation of the carrier-scattering mechanism, have been successfully used to improve the thermoelectric performance of Mg_3_Sb_2-*x*_Bi*_x_* alloys. Further research efforts are warranted to explore other effective and inexpensive dopants for wider temperature application such as in power generation and solid-state cooling, including the structural variation induced by these dopants, and effective strategies to improve thermal stability. In addition, the carrier-scattering mechanism needs to be clarified (whether ionized impurity scattering or grain boundary scattering can better explain the dramatic increase in mobility around room temperature) in the near future in order to further enhance the *zT*. Even so, Mg_3_Sb_2-*x*_Bi*_x_* alloys show great potential for power generation and cooling applications.

## References

[1] He J., Tritt T. M. (2017). Advances in thermoelectric materials research: looking back and moving forward. *Science*.

[2] Mao J., Liu Z., Zhou J. (2018). Advances in thermoelectrics. *Advances in Physics*.

[3] Shuai J., Mao J., Song S., Zhang Q., Chen G., Ren Z. (2017). Recent progress and future challenges on thermoelectric Zintl materials. *Materials Today Physics*.

[4] Zhang Q. H., Huang X. Y., Bai S. Q., Shi X., Uher C., Chen L. D. (2016). Thermoelectric devices for power generation: recent progress and future challenges. *Advanced Engineering Materials*.

[5] Chen X., Wu H., Cui J. (2018). Extraordinary thermoelectric performance in n-type manganese doped Mg_3_Sb_2_ Zintl: high band degeneracy, tuned carrier scattering mechanism and hierarchical microstructure. *Nano Energy*.

[6] Witkoske E., Wang X., Maassen J., Lundstrom M. (2019). Universal behavior of the thermoelectric figure of merit, *zT*, vs. quality factor. *Materials Today Physics*.

[7] Xiao Y., Zhao L.-D. (2018). Charge and phonon transport in PbTe-based thermoelectric materials. *npj Quantum Materials*.

[8] Chen Z., Jian Z., Li W. (2017). Lattice dislocations enhancing thermoelectric PbTe in addition to band convergence. *Advanced Materials*.

[9] Chen Z.-G., Shi X., Zhao L.-D., Zou J. (2018). High-performance SnSe thermoelectric materials: progress and future challenge. *Progress in Materials Science*.

[10] Mao L., Yin Y., Zhang Q. (2020). Fermi-surface dynamics and high thermoelectric performance along the out-of-plane direction in n-type SnSe crystals. *Energy & Environmental Science*.

[11] Luo Y., Zheng Y., Luo Z. (2018). n-type SnSe_2_ oriented-nanoplate-based pellets for high thermoelectric performance. *Advanced Energy Materials*.

[12] Zhu H., Mao J., Li Y. (2019). Discovery of TaFeSb-based half-Heuslers with high thermoelectric performance. *Nature Communications*.

[13] Liu Z., Guo S., Wu Y. (2019). Design of high‐performance disordered half‐Heusler thermoelectric materials using 18‐electron rule. *Advanced Functional Materials*.

[14] Zhao L.-D., Kanatzidis M. G. (2016). An overview of advanced thermoelectric materials. *Journal of Materiomics*.

[15] Shi X., Chen L., Uher C. (2016). Recent advances in high-performance bulk thermoelectric materials. *International Materials Review*.

[16] Zhang J., Song L., Sist M., Tolborg K., Iversen B. B. (2018). Chemical bonding origin of the unexpected isotropic physical properties in thermoelectric Mg_3_Sb_2_ and related materials. *Nature Communications*.

[17] Tamaki H., Sato H. K., Kanno T. (2016). Isotropic conduction network and defect chemistry in Mg_3+*δ*_Sb_2_-based layered Zintl compounds with high thermoelectric performance. *Advanced Materials*.

[18] Shuai J., Wang Y., Kim H. S. (2015). Thermoelectric properties of Na-doped Zintl compound: Mg_3−*x*_Na*_x_*Sb_2_. *Acta Materialia*.

[19] Meng F., Sun S., Ma J., Chronister C., He J., Li W. (2020). Anisotropic thermoelectric figure-of-merit in Mg_3_Sb_2_. *Materials Today Physics*.

[20] Zhang J., Song L., Pedersen S. H., Yin H., Hung L. T., Iversen B. B. (2017). Discovery of high-performance low-cost n-type Mg_3_Sb_2_-based thermoelectric materials with multi-valley conduction bands. *Nature Communications*.

[21] Ohno S., Imasato K., Anand S. (2018). Phase boundary mapping to obtain n-type Mg_3_Sb_2_-based thermoelectrics. *Joule*.

[22] Shuai J., Ge B., Mao J., Song S., Wang Y., Ren Z. (2018). Significant role of Mg stoichiometry in designing high thermoelectric performance for Mg_3_(Sb,Bi)_2_-based n-type Zintls. *Journal of the American Chemical Society*.

[23] Mao J., Zhu H., Ding Z. (2019). High thermoelectric cooling performance of n-type Mg_3_Bi_2_-based materials. *Science*.

[24] Shuai J., Mao J., Song S. (2017). Tuning the carrier scattering mechanism to effectively improve the thermoelectric properties. *Energy & Environmental Science*.

[25] Mao J., Shuai J., Song S. (2017). Manipulation of ionized impurity scattering for achieving high thermoelectric performance in n-type Mg_3_Sb_2_-based materials. *Proceedings of the National Academy of Sciences of the United States of America*.

[26] Imasato K., Kang S. D., Snyder G. J. (2019). Exceptional thermoelectric performance in Mg_3_Sb_0.6_Bi_1.4_ for low-grade waste heat recovery. *Energy & Environmental Science*.

[27] Shi X., Zhao T., Zhang X. (2019). Extraordinary n-type Mg_3_SbBi thermoelectrics enabled by yttrium doping. *Advanced Materials*.

[28] Zhang J., Song L., Iversen B. B. (2020). Rapid one-step synthesis and compaction of high-performance n-type Mg_3_Sb_2_ thermoelectrics. *Angewandte Chemie International Edition*.

[29] Li J., Zhang S., Jia F. (2020). Point defect engineering and machinability in n-type Mg_3_Sb_2_-based materials. *Materials Today Physics*.

[30] Zhang J., Song L., Iversen B. B. (2019). Insights into the design of thermoelectric Mg_3_Sb_2_ and its analogs by combining theory and experiment. *npj Computational Materials*.

[31] Zhang J., Iversen B. B. (2019). Fermi surface complexity, effective mass, and conduction band alignment in n-type thermoelectric Mg_3_Sb_2 – *x*_Bi*_x_* from first principles calculations. *Journal of Applied Physics*.

[32] Shu R., Zhou Y., Wang Q. (2019). Mg_3+*δ*_Sb*_x_*Bi_2−*x*_ family: a promising substitute for the state-of-the-art n-type thermoelectric materials near room temperature. *Advanced Functional Materials*.

[33] Pan Y., Yao M., Hong X. (2020). Mg_3_(Bi,Sb)_2_ single crystals towards high thermoelectric performance. *Energy & Environmental Science*.

[34] Shi X., Sun C., Zhang X. (2019). Efficient Sc-doped Mg_3.05–*x*_Sc*_x_*SbBi thermoelectrics near room temperature. *Chemistry of Materials*.

[35] Zhang J., Song L., Borup K. A., Jørgensen M. R. V., Iversen B. B. (2018). New insight on tuning electrical transport properties via chalcogen doping in n-type Mg_3_Sb_2_-based thermoelectric materials. *Advanced Energy Materials*.

[36] Sun X., Li X., Yang J. (2019). Achieving band convergence by tuning the bonding ionicity in n-type Mg_3_Sb_2_. *Journal of Computational Chemistry*.

[37] Li J., Zhang S., Zheng S. (2019). Defect chemistry for n-type doping of Mg_3_Sb_2_-based thermoelectric materials. *Journal of Physical Chemistry C*.

[38] Li J., Jia F., Zhang S. (2019). The manipulation of substitutional defects for realizing high thermoelectric performance in Mg_3_Sb_2_-based Zintl compounds. *Journal of Materials Chemistry A*.

[39] Gorai P., Toberer E. S., Stevanović V. (2019). Effective n-type doping of Mg_3_Sb_2_ with group-3 elements. *Journal of Applied Physics*.

[40] Gorai P., Ortiz B. R., Toberer E. S., Stevanović V. (2018). Investigation of n-type doping strategies for Mg_3_Sb_2_. *Journal of Materials Chemistry A*.

[41] Chen Y., Wang C., Ma Z., Li L., Li S., Wang J. (2021). Improved thermoelectric performance of n-type Mg_3_Sb_2_-Mg_3_Bi_2_ alloy with Co element doping. *Current Applied Physics*.

[42] Imasato K., Wood M., Kuo J. J., Snyder G. J. (2018). Improved stability and high thermoelectric performance through cation site doping in n-type La-doped Mg_3_Sb_1.5_Bi_0.5_. *Journal of Materials Chemistry A*.

[43] Song S. W., Mao J., Bordelon M. (2019). Joint effect of magnesium and yttrium on enhancing thermoelectric properties of n-type Zintl Mg_3+*δ*_Y_0.02_Sb_1.5_Bi_0.5_. *Materials Today Physics*.

[44] Zhang F., Chen C., Yao H. (2019). High-performance n-type Mg_3_Sb_2_ towards thermoelectric application near room temperature. *Advanced Functional Materials*.

[45] Mao J., Wu Y., Song S. (2017). Anomalous electrical conductivity of n-type Te-doped Mg_3.2_Sb_1.5_Bi_0.5_. *Materials Today Physics*.

[46] Jørgensen L. R., Zhang J., Zeuthen C. B., Iversen B. B. (2018). Thermal stability of Mg_3_Sb_1.475_Bi_0.475_Te_0.05_ high performance n-type thermoelectric investigated through powder X-ray diffraction and pair distribution function analysis. *Journal of Materials Chemistry A*.

[47] Wood M., Imasato K., Anand S., Yang J., Snyder G. J. (2020). The importance of the Mg-Mg interaction in Mg_3_Sb_2_-Mg_3_Bi_2_ shown through cation site alloying. *Journal of Materials Chemistry A*.

[48] Shang H., Liang Z., Xu C. (2020). N-type Mg_3_Sb_2-*x*_Bi*_x_* with improved thermal stability for thermoelectric power generation. *Acta Materialia*.

[49] Zhang J., Song L., Mamakhel A., Jørgensen M. R. V., Iversen B. B. (2017). High-performance low-cost n-type Se-doped Mg_3_Sb_2_-based Zintl compounds for thermoelectric application. *Chemistry of Materials*.

[50] Mao J., Wu Y., Song S. (2017). Defect engineering for realizing high thermoelectric performance in n-type Mg_3_Sb_2_-based materials. *ACS Energy Letters*.

[51] Kuo J. J., Wood M., Slade T. J., Kanatzidis M. G., Snyder G. J. (2020). Systematic over-estimation of lattice thermal conductivity in materials with electrically-resistive grain boundaries. *Energy & Environmental Science*.

[52] Kuo J. J., Kang S. D., Imasato K. (2018). Grain boundary dominated charge transport in Mg_3_Sb_2_-based compounds. *Energy & Environmental Science*.

[53] Kanno T., Tamaki H., Sato H. K. (2018). Enhancement of average thermoelectric figure of merit by increasing the grain-size of Mg_3.2_Sb_1.5_Bi_0.49_Te_0.01_. *Applied Physics Letters*.

[54] Wood M., Kuo J. J., Imasato K., Snyder G. J. (2019). Improvement of low‐temperature *zT* in a Mg_3_Sb_2_–Mg_3_Bi_2_ solid solution via Mg-vapor annealing. *Advanced Materials*.

[55] Shi X., Sun C., Bu Z. (2019). Revelation of inherently high mobility enables Mg_3_Sb_2_ as a sustainable alternative to n‐Bi_2_Te_3_ thermoelectrics. *Advanced Science*.

[56] Kuo J. J., Yu Y., Kang S. D., Cojocaru-Mirédin O., Wuttig M., Snyder G. J. (2019). Mg deficiency in grain boundaries of n-type Mg_3_Sb_2_ identified by atom probe tomography. *Advanced Materials Interfaces*.

[57] Imasato K., Fu C., Pan Y. (2020). Metallic n-type Mg_3_Sb_2_ single crystals demonstrate the absence of ionized impurity scattering and enhanced thermoelectric performance. *Advanced Materials*.

[58] Xin J., Li G., Auffermann G. (2018). Growth and transport properties of Mg_3_*X*_2_ (*X* = Sb, Bi) single crystals. *Materials Today Physics*.

[59] Zhu Q., Song S., Zhu H., Ren Z. (2019). Realizing high conversion efficiency of Mg_3_Sb_2_-based thermoelectric materials. *Journal of Power Sources*.

